# The Detection of Early Pulmonary Tubercle

**Published:** 1908-10-31

**Authors:** 


### THE DETECTION OF EARLY PULMONARY TUBERCLE.

The perennial interest of this subject has been lately shown, both by the large number of those giving a trial to Calmette's ophthalmo-reaction, and by the discussion at the last British Medical Association meeting of a paper by Dr. Leonard, who approached the question from the rontgenological side. On the whole, contemporary opinion has pronounced against the sufficient practical utility of either of these means of diagnosis. Older clinical methods, therefore, still remain pre-eminent. In the last volume of the *Deutsche Klinik*, Krönig shows himself a strong adherent of this view. Diagnoses made from skiagrams and the results of tuberculin injection are, he says, "clinical abortions"—the only true basis being a detailed physical examination. He goes on to give some useful and occasionally novel hints. Inspection of the back of the thorax is more important than that of the front. One should mark out with a crayon the outlines of the scapulæ and the line of the spine, in order to be better able to compare the relative height of the shoulder-blades, and their distance from the mid-line. Even in early tubercle of one apex, the shoulder-blade of the affected side may be lower than its fellow, or displaced a little outwards. These two points are best observed when the patient keeps his thorax as far as may be at rest. On deep breathing, the abnormally placed scapula may be seen to lag behind the other in its movements. In male subjects, or in young girls, the position of the nipples relatively to the ribs should be noted and compared. Palpation is of little service except in the form of gentle direct percussion with the bent tips of the second, third, and fourth fingers. By the sense of touch one may appreciate increased resistance, and although this method is inferior to indirect percussion as regards estimating modifications of resonance, yet it serves as a preliminary, and enables one, as it were, to get one's bearings for the latter. It may also give warning of local tenderness, which last is an important sign. Parenthetically, we may hope that our already exuberant medical terminology will not be increased by his word "Apicalgia." Indirect percussion is especially topographical. One must percuss out the limits of each apex in quiet, and in deep breathing, and compare them. Similarly, the extent of the complemental spaces at the pulmonary bases are to be ascertained. Deficient basal excursion of one lung points to pleural adhesions, which Krönig thinks a very frequent concomitant of even the smallest apical foci. Contrary to the general teaching, auscultation is not to be considered the most important part of the examination, partly because non-tuberculous apical catarrhs are not altogether uncommon, partly because auscultatory signs are variable; adventitious sounds may be heard one day and not the next—may be present in the morning and not in the afternoon, as they probably depend upon the apical retention of secretion. However he condemns the plan of giving potassium iodide in order to set up a little catarrh, and thus make the diseased area manifest. It is bad for a tuberculous patient and open to fallacy, since the healthy side may yield fine rhonchi after such treatment. A method repeatedly tested both by himself and by his assistant with good results, is to prevent the patient coughing and expelling secretion by administering codeine overnight, and examining early next morning. This gives the greatest chance of hearing râles. Increased vocal resonance is best elicited by making the patient whisper; the laryngo-tracheal column of air acts as a fine percussing agent, and the diseased lung, a more homogeneous medium than the healthy one, transmits the sound distinctly to the ear.

